# Biodegradable In Situ Gel-Forming Controlled Drug Delivery System Based on Thermosensitive Poly(**ε**-caprolactone)-Poly(ethylene glycol)-Poly(**ε**-caprolactone) Hydrogel

**DOI:** 10.5402/2012/976879

**Published:** 2012-11-27

**Authors:** Elham Khodaverdi, Ali Golmohammadian, Seyed Ahmad Mohajeri, Gholamhossein Zohuri, Farnaz Sadat Mirzazadeh Tekie, Farzin Hadizadeh

**Affiliations:** ^1^Department of Pharmaceutics, School of Pharmacy, Mashhad University of Medical Sciences, Mashhad, Iran; ^2^Targeted Drug Delivery Research Center, School of Pharmacy, Mashhad University of Medical Sciences, Mashhad, Iran; ^3^Department of Chemistry, Faculty of Science, Ferdowsi University, Mashhad, Iran; ^4^Department of Pharmaceutics, School of Pharmacy, Tehran University of Medical Sciences, Tehran, Iran; ^5^Biotechnology Research Center, School of Pharmacy, Mashhad University of Medical Sciences, Mashhad, Iran

## Abstract

Traditional drug delivery systems which are based on multiple dosing regimens usually pose many disadvantages such as poor compliance of patients and drug plasma level variation. To overcome the obstacles of traditional drug formulations, novel drug delivery system PCL-PEG-PCL hydrogels have been purposed in this study. Copolymers were synthesized by rapid microwave-assisted and conventional synthesis methods. Polymer characterizations were done using gel permeation chromatography and ^1^H-NMR. Phase transition behavior was evaluated by inverting tube method and in vitro drug release profile was determined using naltrexone hydrochloride and vitamin B_12_ as drug models. The results indicated that loaded drug structure and copolymer concentration play critical roles in release profile of drugs from these hydrogels. This study also confirmed that synthesis of copolymer using microwave is the most effective method for synthesis of this kind of copolymer.

## 1. Introduction

Treatment of disease by multiple dosing strategies and using conventional drug formulation poses many drawbacks [1]. Among them are fluctuation of drug plasma concentration, the various side effects due to toxic plasma level of drug, or ineffectiveness treatment because of the lower concentration of drug than its therapeutic index [2, 3]. Another important limitation of multiple dosing regimens is poor patient compliance especially for diseases for which patient must take drugs for a long time such as epilepsy [4, 5] or for some specific drugs such as naltrexone hydrochloride which is used for maintenance abstinence in detoxified addicted persons after withdrawal phase of opioids and alcohol [6, 7].

Nowadays scientists are interested in novel drug delivery systems which are used to direct drugs to the specific site of action and to achieve a controlled release of drug with a desirable release kinetics [1, 8]. Among the most studied of these systems are hydrogels which are insoluble matrices of hydrophilic block copolymers which swell in presence of fluids [9, 10]. Smart hydrogels are the polymeric networks which release drugs in response to the environmental parameters such as temperature [11–13], pH [14, 15], light [16, 17], magnetisms [18, 19]. Thermoresponsive hydrogels are extensively investigated smart hydrogels because of their simple application, low adverse effect on tissues compared with some other stimuli, and the difference between room and body temperature [12, 20].

Amphiphilic triblock copolymers, PCL-PEG-PCL, composed of polyethylene glycol (PEG) and poly (*ε*-caprolactone) (PCL), form a thermoresponsive, in situ forming, and biodegradable hydrogel [21, 22] which have been studied as drug delivery systems [23–25].

 MW irradiation for the high-speed, reproducible, and scalable preparation of polymeric materials has been utilized to reduce reaction times from days and hours to minutes and seconds and facilitate scale-up procedure and technology transfer from bench to industry [26, 27].

In this study, PCL-PEG-PCL with various weight ratios of each block of copolymer (PCL: PEG: PCL), 1000 : 1000 : 1000 and 500 : 1000 : 500, was synthesized by three different methods: using stainless steel reactor, two-necked round-bottomed flask, and under microwave irradiation. Structure, molecular weight (Mw), and sol-gel transition of copolymers were investigated. Also in vitro drug release behavior was evaluated in this study using naltrexone hydrochloride and vitamin B_12_.

## 2. Materials

Poly (ethylene glycol) (PEG, Mn = 1000, Merck, Germany), *ε*-caprolactone (Sigma Aldrich, USA), stannous octoate (Sn(Oct)_2_, Sigma Aldrich, USA), naltrexone hydrochloride was kindly donated by Razak Co., Iran, and vitamin B_12_ were kindly donated by Iran Hormone Co., Iran.

## 3. Methods

### 3.1. Synthesis and Purification of PCL-PEG-PCL Copolymers

Synthesis of PCL-PEG-PCL copolymers was carried out with two different block lengths of (PCL: PEG: PCL): 1000 : 1000 : 1000 (copolymer 1) and 500 : 1000 : 500 (copolymer 2) by a ring-opening polymerization technique. The synthesis in stainless steel reactor and two-necked round-bottomed flask was performed as described before by Gong et al. [21, 28]. The copolymer was also synthesized using microwave irradiation. Briefly to synthesize 1000 : 1000 : 1000, 10 g dried PEG1000, 20 g *ε*-caprolactone, and 2 g Sn(Oct)_2_ were added to a glassy flask fitted with condenser and was placed in a microwave. The mixture was irradiated and stirred at 800 watt while temperature was kept at 130°C. The irradiation time was different in each synthesis (15, 20, 25 min) to choose an optimum time for rapid preparation of the copolymer. 

In order to purify the product, at first, obtained copolymers were dissolved in 20 mL dichloromethane. By gradually adding 800 mL of petroleum ether, the viscous copolymer precipitants appeared. At the final step, the precipitants were filtered. The purification process was done 3 times and the final product was dried by freeze drying method [29]. 

The yield of reaction by each method was calculated by dividing the weight of the purified PCL-PEG-PCL to the weight of initial materials used in the polymer preparation. 

### 3.2. Characterization of the PCL-PEG-PCL Copolymer

The copolymer structure and number-average molecular weights (Mn) were determined using Nuclear Magnetic Resonance Analysis (^1^H-NMR, AC 80 Brucker, Germany) at 80 MHZ and 25°C in CDCL_3_ [21].

Gel permeation chromatography (GPC, Agilent GPC-Addon apparatus and RID-A refractive index signal detector coupled to Plgel columns) was utilized to determine the weight-average molecular weight (Mw), Mn, and polydispersity of the block copolymers using tetrahydrofuran as the solvent and eluent with a rate of 1 mL/min and polystyrene as the calibration standard [29]. 

### 3.3. Evaluation of the Phase Transition of the PCL-PEG-PCL Copolymer

To determine the sol to gel and gel to precipitant transition temperatures, 3 mL of the copolymers solution in distilled water (15, 20, 25, and 30% (w/v)) was poured in to 5 mL vials kept in a refrigerated bath circulator (P-22 WISD, South Korea). The temperature increased with a rate of 1°C/3 min from 10 ± 0.1 to 60 ± 0.1°C. After each 3 min, the vials were inverted to evaluate the viscosity of the content. The temperature which the content did not flow by inverting vials was assumed as the sol-gel transition temperature. An increase in temperature was continued and the precipitation temperature was also recorded [21]. This study was done before and after drug loading to determine the effect of drug on the phase transition temperatures. The reversibility of the gel and the precipitant forming was also investigated by decreasing the temperature. To determine the time of the formation of the gels, all samples were transferred to a 37°C water bath (N-Biotek-304, South Korea) and the time of sol-gel transition was recorded. All experiments were performed 3 times.

### 3.4. In Vitro Drug Release Study

According to the result of the polymer characterizations and the phase transition evaluations, two appropriate concentrations of copolymer 1 (1000-1000-1000), which was synthesized by a microwave irradiation in 15 min, were chosen as a drug delivery system for the in vitro drug release experiment. For the purpose of preparing drug loaded hydrogels, adequate amount of the copolymer was dissolved in a naltrexone hydrochloride or vitamin B_12_ solution in distilled water. The blank was the copolymer solution without drug.  Table 1 presents the different samples that were used in this experiment. The entire samples were prepared in triplicate. 2 mL of each formulation was placed in tubes and was transferred to the reciprocal water bath at 37°. After the formation of the hydrogels, 4 mL of the phosphate buffer solution (PBS, pH = 7.4) was added to each sample tube as a release media and samples were shaken at a rate of 60 times/min. In each sampling time, 0.5 mL of the medium was withdrawn and replaced by 0.5 mL fresh buffer. Drug analysis in samples was performed using high performance liquid chromatography (HPLC, Acme 9000 Young Lin, South Korea, C18 (4.6 × 250 mm, 52 *μ*m) column). Mobile phase for naltrexone hydrochloride was composed of 14% acetonitrile, 85.5% deionized water, and 0.5% acetic acid (v/v) and for B_12_ was a mixture of 30% methanol and 70% deionized water. The flow rate in both analyses was 1 mL/min and the UV detection wave length was set at 281 and 305 nm for naltrexone hydrochloride and B_12_, respectively. 

### 3.5. Statistical Analysis

The results were presented as means ± SDs (*n* = 4). Paired *t*-test and one-way ANOVA were used to analyze them. A significance level was *P* < 0.05 in all cases.

## 4. Results and Discussion

### 4.1. Copolymer Characterizations

In order to compare different copolymer synthesis methods, The copolymerization was done by three different procedures that two of them, using two-necked flask and the stainless steel reactor, were conventional methods and take longer time compared to the other method which was a high-speed microwave-assisted way of the copolymer synthesis. Since the microwave heating process provided a reaction condition with a higher temperature and pressure, the reaction took relatively short times and also the side reaction was limited and consequently greater yields were usually obtained [27]. According to the yield of each method (Table 2), the microwave-assisted synthesis was the best method in which the reaction just took a few minutes. By increasing the time of the reaction from 15 min to 25 min, no significant difference was found in the yield of the reaction or the quality of the products. Thus we considered 15 min as an adequate time for the microwave-assisted synthesis of the PCL-PEG-PCL copolymers. Yields of the copolymers synthesized by the stainless steel reactor method were a little higher than the two-necked flask method.

**Figure 1:**
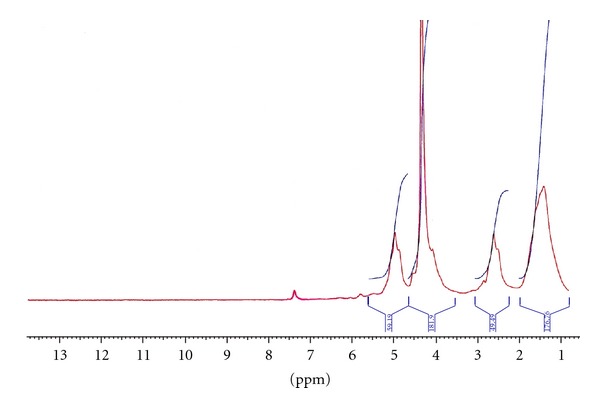
^1^H-NMR spectrum of the tri-block copolymer of PCL-PEG-PCL (1000-1000-1000).

**Figure 2:**
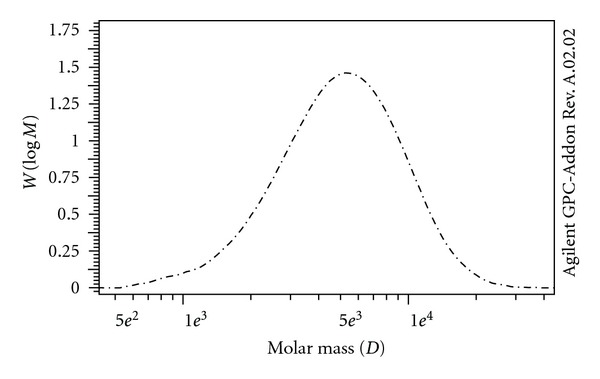
GPC chromatogram of the tri-block copolymer of PCL-PEG-PCL (1000-1000-1000).

The ^1^H-NMR spectrum of the PCL-PEG-PCL (1000-1000-1000) synthesized by the stainless steel reactor is shown in Figure 1. The signals appeared in 3.6 (A), 4.1 (h), 2.2 (d), and 1.5 (e, f, g) ppm are related to the protons of PEG (A) and the CH_2_ groups of caprolactone (h, d, e, f, g) [29]. The Mn of the copolymer was calculated by the integration of the signals pertaining to each monomer. Mn of the copolymer (1000-1000-1000) synthesized by the reactor and the microwave (15 min) were 2590 and 3412, respectively. As the Mn of the copolymers indicated, the one which was prepared by the reactor, in contrast to the one which prepared by the microwave-assisted method, has Mn lower than the theoretical amount (3000).  Figure 2 exhibits the GPC chromatogram of copolymer 1 prepared by the microwave irradiation. Symmetric peak of the chromatogram confirmed the low polydispersity of the copolymer. Mw and Mn obtained from ^1^H-NMR and GPC, the polydispersity of the copolymer which was estimated by Mw/Mn obtained from GPC, and the caprolactone (CL) to PEG ratio obtained from ^1^H-NMR are presented in Table 3. The data in Table 3 belongs to copolymer 1 synthesized by the microwave-assisted method. 

### 4.2. The Phase Transition Behavior

The PCL-PEG-PCL tri-block copolymers are in situ gel-forming thermo-responsive copolymers composed of the hydrophobic PCL and the hydrophilic PEG blocks. When hydrophobic blocks of PCL are surrounded by water molecules, they aggregate to reduce their exposed surface area in an aqueous environment and form micelles. But this phenomenon is temperature dependent. In temperatures below (lower critical solution temperature) LCST, since there is hydrogen bonding between hydrophilic PEG blocks and water molecules, the system is in sol condition and contains micelles. By increasing the temperature above LCST, weakening of the hydrogen bonds and the formation of the hydrophobic bridge between micelles through PCL segments cause the system to become gel. If increasing the temperature continues, the copolymers are phased out [29].

The sol-gel transition temperatures of the copolymers with various concentrations are presented in Table 4. Copolymer 1 (1000-1000-1000) could form gel in all concentrations, but copolymer 2 (500-1000-500) prepared by both conventional synthesis method could not form gel at the concentration of 15% and not even at the concentration of 20% for the two-necked flask method (w/v) due to its shorter hydrophobic segments (CL) compared to copolymer 1 and an incomplete copolymer synthesis by these methods. Although the copolymer 1 (1000-1000-1000) formed gel in all concentrations, the sol-gel transition occurred in lower temperatures when it was synthesized using the microwave irradiation (*P* < 0.05). In all methods, as the copolymer concentrations increased, the phase transition temperatures shifted to the lower temperatures due to an increase in hydrophobic interactions. There was no significant difference between the gel forming temperatures of the copolymers synthesized by the microwave method in different reaction times (*P* > 0.05). The phase transition temperatures in all concentrations of copolymer 1 synthesized by the microwave irradiation were below the room temperature. This causes copolymer 1 synthesized by the microwave to become a good candidate for an injectable carrier which converts to the gel in the body and releases the drug loaded inside in a sustained and controlled manner. The phase transition temperatures of all copolymers synthesized by the conventional methods were above the body temperature and therefore could not gel in the body and were not suitable as a drug carrier.

Another limitation of the conventional synthesized copolymers was the delay in gel formation that may cause a dose dumping effect in the body. For the microwave-assisted synthesized copolymers, the sol-gel transition occurred in about 20 seconds, whereas this amount was 20 min for some of the copolymers synthesized by the conventional methods. 

**Figure 3:**
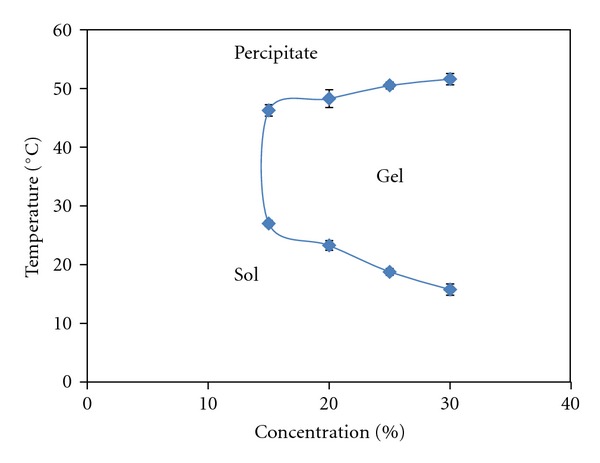
Effect of the PCL-PEG-PCL (1000-1000-1000) concentrations on the phase transition temperature.

The effect of the copolymer concentration on the phase transition temperature was shown in Figure 3. As it is indicated, by increasing copolymer 1 concentration (microwave synthesized), the sol-gel transition temperatures decreased and the precipitation temperatures increased due to a larger number of hydrophobic interactions. 

We also investigated the effect of the drug content on the sol-gel transition temperature. As it was indicated in Table 5, loading vitamin B_12_, a large molecule having 9 positions on it that can form hydrogen bonds with water molecules, lowered the activity of water and favored hydrophobic interactions between the PCL blocks of the copolymers. However, loading a fairly small molecule such as naltrexone hydrochloride in copolymers does not have a significant effect on the phase transition temperatures.

### 4.3. In Vitro Drug Release Study

**Figure 4:**
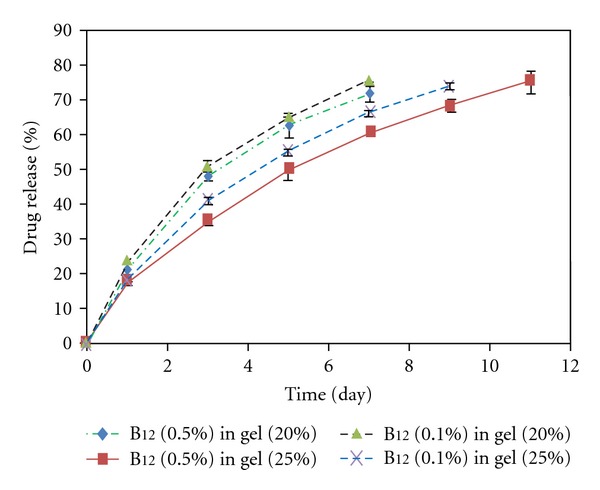
B_12_ release from copolymer (1000-1000-1000) with different concentration of drug and polymers.

**Figure 5:**
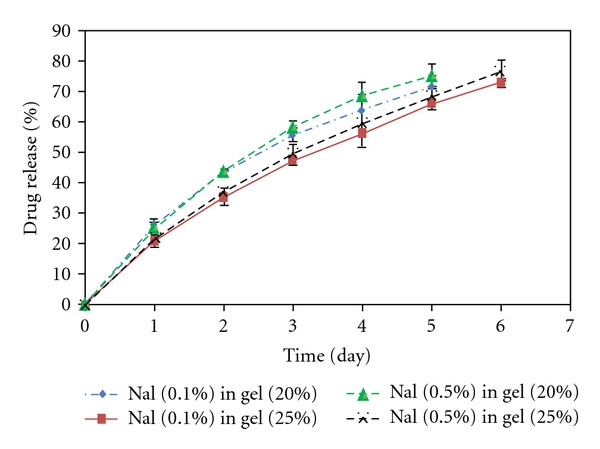
Naltrexone hydrochloride (Nal) release from copolymer (1000-1000-1000) with different concentration of drug and polymers.

**Figure 6:**
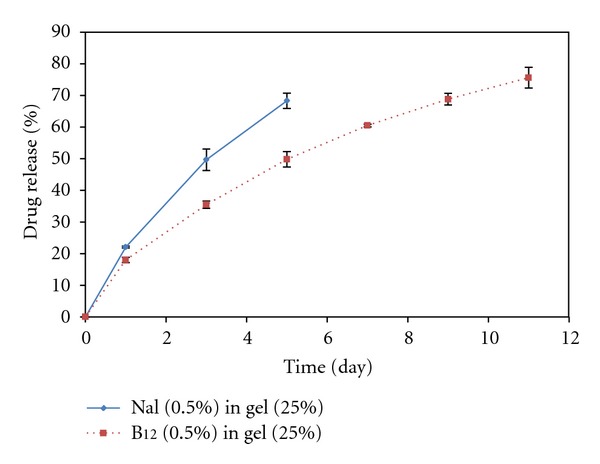
Comparison between the release profile of naltrexone hydrochloride (Nal, 0.5% (w/v)) and B_12_ (0.5% (w/v)) through the hydrogels with 25% (w/v) copolymer.

In vitro drug release through the PCL-PEG-PCL (1000-1000-1000) synthesized by the microwave-assisted method was investigated at different concentrations of the copolymer and the drugs. The drug models used in this experiment were naltrexone hydrochloride and vitamin B_12_. The results are presented in Figures 4–6. The rate of drug release through the hydrogels with the same concentration of the copolymer but a different concentration of the drug was not significant; just a negligible increase in the rate of drug release after an increase in naltrexone hydrochloride concentration was observed. This could be due to the fact that naltrexone hydrochloride, which is a small molecule in contrast to B_12_, was released by diffusion rather than the hydrogel degradation. According to the Fick's law of diffusion, increasing drug concentration causes the rate of diffusion and the consequent drug release increases [30]. As it is shown in Figures 4 and 5, the release of naltrexone hydrochloride and vitamin B_12_ through the hydrogels with higher copolymer concentration was slower. This could be attributed to the fact that higher copolymer concentration made the hydrogels low porous with a big tortuosity and viscosity. Here, the effect of the molecular weight on the drug release through the hydrogels was also investigated by comparing the naltrexone hydrochloride and vitamin B_12_ loaded systems. As indicated in Figure 6, vitamin B_12_ with a higher molecular weight (MW = 1355) compared to naltrexone hydrochloride (MW = 373), was released more slowly than naltrexone hydrochloride. The release kinetics of both drugs was determined by fitting data according to zero order (release by hydrogel erosion) and Higuchi kinetic models [31, 32]. Since *R* ^2^ of both models were approximately similar in all formulations, it was assumed that both mechanisms of diffusion and the hydrogel erosion played an important role in the drug release through the hydrogels (Table 6). 

## 5. Conclusion

Synthesis of PCL-PEG-PCL using microwave irradiation is the fast, simple, and cost effective method which causes higher yield of reaction. PCL 1000-PEG 1000-PCL 1000 tri-block copolymer solution which forms gel in body temperature even with low concentration of polymer appears to be a suitable in situ forming system as the pharmaceutical and clinical purpose. Different parameters such as polymer concentration and drug structure have influence on profile of drug release from systems. The release kinetics is mix of diffusion and hydrogel degradation. 

## Figures and Tables

**Table 1:** Samples used in the in vitro drug release study.

No.	Concentration of the B_12_ solution(w/v %)	Concentration of the copolymer solution (w/v %)	No.	Concentration of the naltrexone hydrochloride solution (w/v %)	Concentration of the copolymer solution (w/v %)
1	0	20	11	0	20
2	0	25	12	0	25
3	0.1	20	13	0.1	20
4	0.1	25	14	0.1	25
5	0.5	20	15	0.5	20
6	0.5	25	16	0.5	25

**Table 2:** Comparison of different synthesis methods based on the time and the yield of the reaction.

Kind of the copolymer	Synthesis method	Time of the reaction	Yield of the reactions (%)
Copolymer 1	Two-necked flask	6 hours	56
Copolymer 2	Two-necked flask	6 hours	68
Copolymer 1	Stainless steel reactor	6 hours	73
Copolymer 2	Stainless steel reactor	6 hours	79
Copolymer 1	Microwave irradiation	25 min	93
Copolymer 1	Microwave irradiation	15 min	95
Copolymer 1	Microwave irradiation	20 min	96

**Table 3:** The triblock copolymer 1 (1000-1000-1000) composition determined by GPC and ^1^H NMR.

GPC			NMR	
Mw/Mn	Mw	Mn	CL/PEG	Mn
1.49	5895	3936	2.412	1206-1000-1206

**Table 4:** Sol-gel transition temperatures of different PCL-PEG-PCL copolymers (°C).

Conc. (w/v %)	copolymer 1 microwave 25 min	copolymer 1 microwave 20 min	copolymer 1 microwave 15 min	copolymer 1 reactor	copolymer 2 reactor	copolymer 1 two-necked flask	copolymer 2 two-necked flask
15%	26.25 ± 0.5	27 ± 0	26.5 ± 0.58	53 ± 1.83	No Gel	56.25 ± 1.2	No Gel
20%	23 ± 0.82	23.25 ± 0.5	23.25 ± 0.5	49.75 ± 1.5	55 ± 2.16	50.75 ± 0.9	No Gel
25%	18.5 ± 0.58	18.75 ± 0.5	18.75 ± 0.9	45.5 ± 1.73	53 ± 1.41	45.25 ± 0.5	54.75 ± 0.9
30%	15.75 ± 0.9	15.5 ± 0.58	15.25 ± 1.2	42 ± 1.41	48.5 ± 0.5	43.75 ± 0.7	51.25 ± 0.9

**Table 5:** The effect of drug loading on the sol-gel transition temperature.

Copolymer concentration (w/v %)	sol-gel transition temperature without drug	sol-gel transition temperature B_12_ (0.1%)	sol-gel transition temperature B_12_ (0.5%)	sol-gel transition temperature naltrexone HCl (0.1%)	sol-gel transition temperature naltrexone HCl (0.5%)
20%	23.25 ± 0.5	21.67 ± 0.58	21.33 ± 0.58	22.67 ± 0.58	21.67 ± 0.58

25%	18.75 ± 0.96	18 ± 1	16.33 ± 0.58	18.33 ± 0.58	18 ± 0

**Table 6:** Kinetic profile of drug release.

Drug	Drug concentration (w/v %)	PCL-PEG-PCL concentration (w/v %)	Zero order	Higuchi
			*R* ^2^	*R* ^2^
Naltrexone hydrochloride	0.1	20	0.938	0.993
		25	0.966	0.982
	0.5	20	0.951	0.985
		25	0.966	0.983

Vitamin B_12_	0.1	20	0.928	0.993
		25	0.942	0.993
	0.5	20	0.934	0.990
		25	0.946	0.994

## References

[1] Gupta P, Vermani K, Garg S (2002). Hydrogels: from controlled release to pH-responsive drug delivery. *Drug Discovery Today*.

[2] Sood A, Panchagnula R (2003). Design of controlled release delivery systems using a modified pharmacokinetic approach: a case study for drugs having a short elimination half-life and a narrow therapeutic index. *International Journal of Pharmaceutics*.

[3] Sandberg A, Blomqvist I, Jonsson UE, Lundborg P (1988). Pharmacokinetic and pharmacodynamic properties of a new controlled-release formulation of metoprolol: a comparison with conventional tablets. *European Journal of Clinical Pharmacology*.

[4] Claxton AJ, Cramer J, Pierce C (2001). A systematic review of the associations between dose regimens and medication compliance. *Clinical Therapeutics*.

[5] Cramer JA, Glassman M, Rienzi V (2002). The relationship between poor medication compliance and seizures. *Epilepsy and Behavior*.

[6] Gueorguieva R, Wu R, Pittman B (2007). New insights into the efficacy of naltrexone based on trajectory-based reanalyses of two negative clinical trials. *Biological Psychiatry*.

[7] Rothenberg JL, Sullivan MA, Church SH (2002). Behavioral naltrexone therapy: an integrated treatment for opiate dependence. *Journal of Substance Abuse Treatment*.

[8] He C, Kim SW, Lee DS (2008). In situ gelling stimuli-sensitive block copolymer hydrogels for drug delivery. *Journal of Controlled Release*.

[9] Lin C-C, Metters AT (2006). Hydrogels in controlled release formulations: network design and mathematical modeling. *Advanced Drug Delivery Reviews*.

[10] Klouda L, Mikos AG (2008). Thermoresponsive hydrogels in biomedical applications. *European Journal of Pharmaceutics and Biopharmaceutics*.

[11] Lee JW, Hua F-J, Lee DS (2001). Thermoreversible gelation of biodegradable poly(*ε*-caprolactone) and poly(ethylene glycol) multiblock copolymers in aqueous solutions. *Journal of Controlled Release*.

[12] Ruel-Gariépy E, Leroux J-C (2004). In situ-forming hydrogels—review of temperature-sensitive systems. *European Journal of Pharmaceutics and Biopharmaceutics*.

[13] Khodaverdi E, Rajabi O, Farhadi F, Jalali A, Mirzazadeh Tekie FS (2010). Preparation and investigation of poly (N-isopropylacrylamide-acrylamide) membranes in temperature responsive drug delivery. *Iranian Journal of Basic Medical Sciences*.

[14] Khodaverdi E, Rajabi O, Abdekhodai MJ, Wu XY (2008). Heterogenous composite membranes as pH responsive drug delivery systems. *Iranian Journal of Basic Medical Sciences*.

[15] Albarran B, Hoffman AS, Stayton PS (2011). Efficient intracellular delivery of a pro-apoptotic peptide with a pH-responsive carrier. *Reactive and Functional Polymers*.

[16] Knežević NŽ, Trewyn BG, Lin VSY (2011). Light- and pH-responsive release of doxorubicin from a mesoporous silica-based nanocarrier. *Chemistry*.

[17] Choi SK, Verma M, Silpe J, Moody RE, Tang K, Hanson JJ (2012). A photochemical approach for controlled drug release in targeted drug delivery. *Bioorganic & Medicinal Chemistry*.

[18] Meenach SA, Hilt JZ, Anderson KW (2010). Poly(ethylene glycol)-based magnetic hydrogel nanocomposites for hyperthermia cancer therapy. *Acta Biomaterialia*.

[19] Satarkar NS, Hilt JZ (2008). Magnetic hydrogel nanocomposites for remote controlled pulsatile drug release. *Journal of Controlled Release*.

[20] Zhang X-Z, Zhuo R-X, Cui J-Z, Zhang J-T (2002). A novel thermo-responsive drug delivery system with positive controlled release. *International Journal of Pharmaceutics*.

[21] Gong C, Shi S, Wu L (2009). Biodegradable in situ gel-forming controlled drug delivery system based on thermosensitive PCL-PEG-PCL hydrogel. Part 2: sol-gel-sol transition and drug delivery behavior. *Acta Biomaterialia*.

[22] Wei X, Gong C, Gou M (2009). Biodegradable poly(*ε*-caprolactone)-poly(ethylene glycol) copolymers as drug delivery system. *International Journal of Pharmaceutics*.

[23] Gou M, Dai M, Li X (2008). Preparation of mannan modified anionic PCL-PEG-PCL nanoparticles at one-step for bFGF antigen delivery to improve humoral immunity. *Colloids and Surfaces B*.

[24] Gou M, Zheng L, Peng X (2009). Poly(*ε*-caprolactone)-poly(ethylene glycol)-poly(*ε*-caprolactone) (PCL-PEG-PCL) nanoparticles for honokiol delivery in vitro. *International Journal of Pharmaceutics*.

[25] Zhang L, He Y, Yu M, Song C (2011). Paclitaxel-loaded polymeric nanoparticles based on PCL-PEG-PCL: preparation, *in vitro* and *in vivo* evaluation. *Journal of Controlled Release*.

[26] Dong X, Qi R, Huang Y, Jing X (2011). Synthesis of biodegradable dextran-g-(PCL-b-PEG) by combination of ring-opening polymerization and click chemistry. *Journal of Controlled Release*.

[27] Sosnik A, Gotelli G, Abraham GA (2011). Microwave-assisted polymer synthesis (MAPS) as a tool in biomaterials science: how new and how powerful. *Progress in Polymer Science*.

[28] Gong C, Shi S, Dong P (2009). Synthesis and characterization of PEG-PCL-PEG thermosensitive hydrogel. *International Journal of Pharmaceutics*.

[29] Gong CY, Shi S, Dong PW (2009). Biodegradable in situ gel-forming controlled drug delivery system based on thermosensitive PCL-PEG-PCL hydrogel: part 1—synthesis, characterization, and acute toxicity evaluation. *Journal of Pharmaceutical Sciences*.

[30] Kalia YN, Guy RH (2001). Modeling transdermal drug release. *Advanced Drug Delivery Reviews*.

[31] Higuchi T (1961). Rate of release of medicaments from ointment bases containing drugs in suspension. *Journal of Pharmaceutical Sciences*.

[32] Kim SW, Bae YH, Okano T (1992). Hydrogels: swelling, drug loading, and release. *Pharmaceutical Research*.

